# Angiotensin-converting enzyme genotype and late respiratory complications of mustard gas exposure

**DOI:** 10.1186/1471-2466-8-15

**Published:** 2008-08-14

**Authors:** Ali Reza Hosseini-khalili, Julian Thompson, Anthony Kehoe, Nicholas S Hopkinson, A Khoshbaten, Mohammad Reza Soroush, Steve E Humphries, Hugh Montgomery, Mostafa Ghanei

**Affiliations:** 1 UCL Institute for Human Health and Performance, Ground Floor, Charterhouse Building, UCL Archway Campus, Highgate Hill, Archway, London N19 5LW, UK; 2 Royal Brompton Hospital, Fulham Rd, London SW3 6NP, UK; 3 Janbazan Medical and Engineering Research Center, Tehran, Iran; 4 UCL Cardiovascular Genetics, British Heart Foundation Laboratories, Rayne Inst, 5 University Street, London WC1E 6JJ, UK; 5 Research Center of Chemical Injuries, Baqiyatallah Medical Science University, Mollasadra Street, Tehran, 14359151371, Iran

## Abstract

**Background:**

Exposure to mustard gas frequently results in long-term respiratory complications. However the factors which drive the development and progression of these complications remain unclear. The Renin Angiotensin System (RAS) has been implicated in lung inflammatory and fibrotic responses. Genetic variation within the gene coding for the Angiotensin Converting Enzyme (ACE), specifically the Insertion/Deletion polymorphism (I/D), is associated with variable levels of ACE and with the severity of several acute and chronic respiratory diseases. We hypothesized that the ACE genotype might influence the severity of late respiratory complications of mustard gas exposure.

**Methods:**

208 Kurdish patients who had suffered high exposure to mustard gas, as defined by cutaneous lesions at initial assessment, in Sardasht, Iran on June 29 1987, underwent clinical examination, spirometric evaluation and ACE Insertion/Deletion genotyping in September 2005.

**Results:**

ACE genotype was determined in 207 subjects. As a continuous variable, FEV_1 _% predicted tended to be higher in association with the D allele 68.03 ± 20.5%, 69.4 ± 21.4% and 74.8 ± 20.1% for II, ID and DD genotypes respectively. Median FEV_1 _% predicted was 73 and this was taken as a cut off between groups defined as having better or worse lung function. The ACE DD genotype was overrepresented in the better spirometry group (Chi^2 ^4.9 p = 0.03). Increasing age at the time of exposure was associated with reduced FEV_1 _%predicted (p = 0.001), whereas gender was not (p = 0.43).

**Conclusion:**

The ACE D allele is associated with higher FEV_1 _% predicted when assessed 18 years after high exposure to mustard gas.

## Background

Some 100,000 Iranians were exposed to chemical warfare agents during the 8-year Iraq-Iran war, with the approximately 50,000 mustard gas-affected individuals exhibiting a pattern of late respiratory, eye and skin complications [1]. Chronic bronchitis, asthma, bronchiectasis and pulmonary fibrosis account for the most frequent long-term respiratory sequelae, with progressive decline occurring over many years [2-5].

Mustard gas, bis (2- chloroethyl) sulphide, is a bifunctional alkylating agent. It is a potent vesicant, whose rapid penetration leads to extensive blistering in all epithelial tissues exposed to it. The immediate toxicity of mustard gas is thought to be due to the consequences of both DNA and protein alkylation reactions [6]. However, the factors which drive the development and progression of the long term respiratory complications remain unclear.

The circulating or endocrine renin-angiotensin system (RAS) plays a key role in circulatory homeostasis. Angiotensin Converting Enzyme (ACE) converts angiotensin I to the potent vasoconstrictor angiotensin II. However, local RAS exist in diverse human tissues, where they play proinflammatory and profibrotic roles [7-9]. A lung RAS is now known to exist and is implicated in the genesis of lung inflammatory and fibrotic responses [10].

Whether in the circulation or the tissues [11,12], the absence (deletion, D allele) rather than the presence (insertion, I allele) of a 287 base pair fragment in the human ACE gene is associated with increased ACE activity. In keeping with the postulated roles for ACE in the lung, the D-allele has been associated with development of the acute respiratory distress syndrome, and poorer markers of respiratory function in acute illness [13,14]. However, such detrimental impact of the D-allele in *acute *illness might be counterbalanced by more positive systemic affects in *chronic *disease, as suggested by the association of the D-allele with preserved skeletal muscle strength amongst patients with chronic pulmonary disease [15].

We thus postulated that ACE genotype might influence the severity of the late respiratory complications of mustard gas exposure, and have tested this hypothesis in a pilot study.

## Methods

Thus study was approved by the Ethics Committee of the Iranian Janbazan [Veterans'] organization. Written informed consent was obtained from each subject.

### Subjects

All were Kurdish civilians and had suffered high-level mustard gas exposure at Sardasht, Iran on June 29 1987, as recorded by the Janbazan veterans' organization – the official center for compensation of war disabled victims. In keeping with accepted methodologies, those with only erythema were defined as "low exposure", whilst those with erythema and edema, vesiculation, scaling, ulceration, or crusting are categorized as "high exposure" [16]. Using such criteria, 'high exposure' subjects were selected. Patients were excluded if they had a history of smoking, heart failure, occupational history of chemical agent exposure or a lung inflammatory disorder of a different origin.

### Pulmonary impairment

Spirometric evaluation was performed in September 2005, with forced expiratory volume over 1 second (FEV_1_), forced vital capacity (FVC), and the ratio between them calculated (Multi-Functional Spirometer HI-801, Chest M.I., INC, Tokyo, Japan). Spirometry was performed according to international guidelines with the best of three readings recorded.

### Genotyping

Five milliliters of EDTA blood was obtained from each subject, and ACE genotype was determined using three-primer polymerase chain reaction amplification (PCR) and subsequent agarose gel electrophoresis, as previously reported [17]. All gels included a positive heterozygous control sample, and genotypes were read by two independent observers blind to case/control status. Discrepancies were resolved by repeat PCR.

### Statistical Analysis

Analysis was performed using Statview 5.0 (Abacus concepts, Inc., Berkeley, CA, USA). Deviation from Hardy-Weinberg equilibrium was considered using a chi-squared test. Disease severity was defined according to a median split of FEV_1 _% predicted into better or worse lung function groups. The influence of age, gender and ACE genotype on disease severity was tested using binary logistic regression analysis comparing D homozygotes to other genotypes. Throughout, a p-value < 0.05 was considered statistically significant. Data was normally distributed and results presented as mean ± standard deviation (SD).

## Results

### Patient Recruitment

Overall, 208 patients were enrolled with a mean age of 46.6 ± 14.4 years, of whom 89 (43%) were female (Table 1). Mean FEV_1 _% predicted and FVC % predicted were 70.7 ± 21.0 and 74.4 ± 19.3 respectively. Nearly all reported some respiratory symptoms: 201 (97%) reported dyspnea, 160 (77%) reported chronic cough, 102 (49%) hemoptysis, and 128 (61.5%) chronic sputum production. On physical examination 50 (24%) had an expiratory wheeze, and 12 (5.8%) inspiratory crackles.

**Table 1:** Median split group characteristics

	Mean age	Mean FEV1 % predicted	Mean FVC % predicted	Gender
All subjects (208)	46.6 ± 14.4	70.7 ± 21.0	74.4 ± 19.3	89 (43%) female
FEV_1 _> 73	43.2 ± 13.2	86.2 ± 10.9	88.6 ± 13.9	49 (45.8%) female
FEV_1 _< 73	50.4 ± 14.7	53.9 ± 15.8	62.3 ± 16.9	40 (40%) female

For technical reasons, ACE genotype could not be determined in 1 subject. In the remaining 207 subjects, ACE genotype distribution was 37 (17.9%) vs 115 (55.6%) vs 55 (26.6%) for II, ID and DD respectively, and was consistent with Hardy-Weinberg equilibrium (p value = 0.11). Neither age nor gender varied significantly between ACE genotypes.

**Figure 1:**
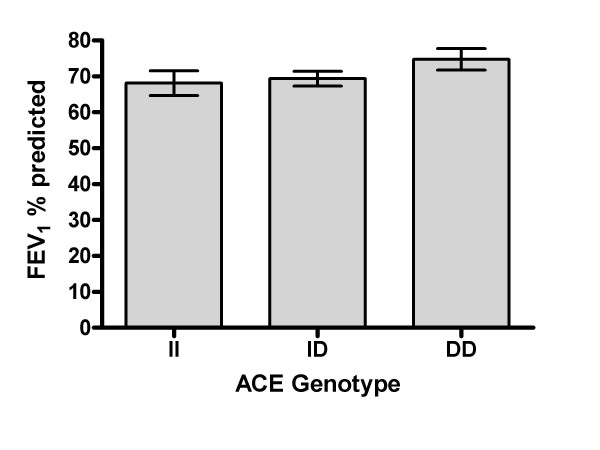
**Mean FEV1 % predicted by ACE genotype**. Error bars represent Standard error mean (SEM). Test for linear trend p = 0.1.

As a continuous variable, FEV_1 _% predicted tended to be higher in association with the D allele 68.03 ± 20.5%, 69.4 ± 21.4% and 74.8 ± 20.1% for II, ID and DD genotypes respectively (Figure 1) linear trend p = 0.10. Analysis of FVC % predicted demonstrated higher values for the DD genotype with FVC % predicted of 74.59 ± 17.2%, 72.9 ± 19.5% and 77.13 ± 20.3% for II, ID and DD genotypes respectively (ANOVA p = 0.43)

Gender did not appear to have an influence on disease severity. Mean FEV_1 _% predicted for males was 71.7 ± 21 and for females 70.0 ± 21. There was no significant association between gender and severity grouping (Chi^2 ^p = 0.43).

Increasing age was associated with reduced FEV_1 _% predicted (r^2 ^0.05 p = 0.001). Mean age in the high FEV_1 _% predicted group was 43.2 ± 13.2 in comparison with 50.4 ± 14.7 in the low FEV_1 _% predicted group (t test p = 0.0002).

Median FEV_1 _% predicted was 73 and this was taken as a cut off between groups demonstrating higher or lower spirometry readings. (Table 1).

The ACE DD genotype was overrepresented in the better spirometry group (Chi^2 ^4.9 (p = 0.03) (Table 2). In a logistic regression model including age this became more significant (p = 0.011).

**Table 2:** Median split groups and ACE genotype

	ACE II	ACE ID	ACE DD	
FEV_1 _> 73	13 (12%)	58 (54%)	36 (33.6%)	107
FEV_1 _< 73	24 (24%)	57 (57%)	19 (19%)	100
All subjects	37 (17.9%)	115 (55.6%)	55 (26.6%)	207

The presence or absence of particular symptoms and signs, or the number of them present was not associated with genotype.

## Discussion

This study is the first to suggest an association between the Angiotensin Converting Enzyme Insertion/Deletion polymorphism and the severity of late respiratory complications of mustard gas exposure, as measured by FEV_1_. 33.6% of subjects in the better lung function group carry the DD-genotype, whereas only 12% in this group carry the II-genotype.

The finding that the D-allele was over-represented in subjects with less severe late pulmonary impairment following exposure to mustard gas might be considered unexpected given the higher ACE activity associated with it and the recognized pro-inflammatory and pro-fibrotic roles of increased RAS activity in the lung. In contrast to this finding, previous studies have demonstrated associations of the D-allele with increased severity of pulmonary sarcoid [18], acute lung injury responses in adults [19], non-infectious pulmonary complications of bone marrow transplantation [20] and of esophageal surgery [21], and with the development of bronchopulmonary dysplasia after premature birth [22].

Recently a study of transbronchial lung biopsies in patients with severe mustard gas related lung disease has revealed histopathological changes diagnosable as organizing pneumonia [3,23]. Previously fibrosis had been considered to be the predominant pathology in the late respiratory complications of mustard gas exposure. The pathogenesis of organizing pneumonia may interact with the RAS in a different way to the generation of fibrosis and help explain the association of the D-allele with less severe pulmonary impairment.

Most studies examining the role of the RAS in lung inflammation and fibrosis have associated the I-allele with less severe disease in the short term response to a pathological insult. This study examined pulmonary impairment nearly 20 years after exposure to mustard gas and may suggest that the RAS has a different influence on chronic pulmonary disease.

Additionally we found the severity of pulmonary impairment following mustard gas exposure to be associated with increasing age – findings that accord with those of Zarchi et al, in a study of 1337 soldiers exposed to mustard gas [24]. The mechanism underlying this increased susceptibility with age is unclear. It could represent a greater susceptibility at a pulmonary level or could be that removal of children from exposure (both physically and the removal of clothing to prevent ongoing exposure) may have been more effective

A further study is warranted – and one which not only includes far greater numbers, but which obtains far greater phenotypic detail – including detailed non-invasive pulmonary function testing, and imaging.

These findings, and the issue of further study, are of importance for a number of reasons. Certainly, gene-environment studies such as this may prove powerful in exploring the fundamental mechanisms driving progressive lung pathology of diverse origin. In addition, however, there are many tens of thousands of patients suffering the pulmonary sequelae of mustard gas exposure, for whom no specific therapeutic modality is yet available. The demonstration that ACE influences such pathology may open the way to new therapeutic options.

## Conclusion

ACE genotype influences the severity of the late respiratory complications of mustard gas exposure with the D allele being associated with higher FEV_1 _% predicted 18 years after exposure.

## Abbreviations

ACE: Angiotensin Converting Enzyme; D: Deletion; FEV_1_ Forced Expiratory Volume over 1 second; FVC: Forced vital capacity; I: Insertion; PCR: Polymerase chain reaction amplification; RAS: Renin Angiotensin System; SD: Standard deviation; SEM: Standard error mean.

## Competing interests

Authors Dr Julian Thompson and Dr Ali Reza Hosseini Khalili; Dr Anthony Kehoe, Dr Nicholas S Hopkinson, Professor A Khoshbaten, Dr Mohammad Reza Soroush, Professor Steve Humphries; Dr Hugh Montgomery and Professor Mostafa Ghanei have no competing interests to disclose.

## Authors' contributions

JT, ARH–K, AKe, NSH, HM drafted the manuscript and performed the statistical analysis. AKh, MRS, SEH, HM and MG conceived of the study, and participated in its design, data acquisition and coordination. All authors read and approved the final manuscript.

## Pre-publication history

The pre-publication history for this paper can be accessed here:


